# Malnutrition drives infection susceptibility and dysregulated myelopoiesis that persists after refeeding intervention

**DOI:** 10.1101/2024.08.19.608575

**Published:** 2024-08-19

**Authors:** Alisa Sukhina, Clemence Queriault, Elise Hall, Kelly Rome, Muskaan Aggarwal, Elizabeth Nunn, Ashley Weiss, Janet Nguyen, Will Bailis

**Affiliations:** 1 Department of Pathology and Laboratory Medicine, Children’s Hospital of Philadelphia; Philadelphia, PA 19104.; 2 Department of Pathology and Laboratory Medicine, Perelman School of Medicine, University of Pennsylvania; Philadelphia, PA 19104.; 3 Institute for Diabetes, Obesity, and Metabolism, Perelman School of Medicine at the University of Pennsylvania, Philadelphia, PA, USA; Department of Physiology, Perelman School of Medicine at the University of Pennsylvania, Philadelphia, PA, USA.

## Abstract

Undernutrition is one of the largest persistent global health crises, with nearly 1 billion people facing severe food insecurity. Infectious disease represents the main underlying cause of morbidity and mortality for malnourished individuals, with infection during malnutrition representing the leading cause of childhood mortality worldwide. In the face of this complex challenge, simple refeeding protocols have remained the primary treatment strategy. Although an association between undernutrition and infection susceptibility has been appreciated for over a century, the underlying mechanisms remain poorly understood and the extent to which refeeding intervention is sufficient to reverse nutritionally acquired immunodeficiency is unclear. Here we investigate how malnutrition leads to immune dysfunction and the ability of refeeding to repair it. We find that chronic malnutrition severely impairs the ability of animals to control a sub-lethal bacterial infection. Malnourished animals exhibit blunted immune cell expansion, impaired immune function, and accelerated contraction prior to pathogen clearance. While this defect is global, we find that myelopoiesis is uniquely impacted, resulting in in reduced neutrophil and monocyte numbers prior to and post-infection. Upon refeeding, we observe that animals recover body mass, size, cellularity across all major immune organs, the capacity to undergo normal immune cell expansion in response to infection, and a restoration in T cell responses. Despite this broad improvement, refed animals remain susceptible to bacterial infection, uncoupling global lymphoid atrophy from immunodeficiency. Mechanistically, we find peripheral neutrophil and monocyte numbers fail to fully recover and refed animals are unable to undergo normal emergency myelopoiesis. Altogether, this work identifies a novel cellular link between prior nutritional state and immunocompetency, highlighting dysregulated myelopoiesis as a major driver. We believe these findings illustrate how exposure to food scarcity is an immunologic variable, even post-recovery, which should be accounted for in patient medical history and current global public health policy.

## Introduction

Almost 500 million adults and over 200 million children are affected by undernutrition worldwide, with over half of all childhood deaths linked to undernutrition (United Nations, 2021; UNICEF, 1998). In the past 5 years, these numbers worsened due to economic instability ushered by COVID-19 pandemic, and they are projected to further increase throughout the impending climate crisis (Mbow et al., 2019; United Nations, 2022). In undernourished patients, the largest contributor to morbidity and mortality is infectious disease (Fan et al., 2022; Rice et al., 2000). It has been demonstrated that undernutrition alone puts patients, especially children, at a higher risk of developing long-lasting disability or dying from an infection (Antwi 2011; Bhargava, 2016; Bourke et al., 2016; Caulfield et al., 2004; Rice et al., 2000; Schlaudecker et al., 2011; Sinha et al., 2021). In addition to increased susceptibility to infection, chronically undernourished individuals have been reported to have impaired barrier function, atrophy of immune organs, and less effective responses to vaccines (Bhattacharjee et al., 2021; Bourke et al., 2016; Collins and Belkaid, 2020; Prendergast, 2015). In keeping with this, undernutrition is the leading cause of secondary immunodeficiency in the world (Chinen and Shearer, 2010). Considering the geographical overlap between areas with high prevalence of undernutrition and infectious disease and the persistent nature of the global undernutrition crisis, it is critical to investigate how undernutrition causes immunodeficiency and contributes to poor infection outcomes (Mbow et al., 2019; Murray et al., 1996; Pelletier, 1994; Roberts, 2017; Rohr et al., 2019; Sinha et al., 2021; UNICEF-WHO-World Bank JME Working Group, 2021).

Despite the first link between undernutrition and poor infection outcomes being posited over a century ago, the cellular mechanisms underlying undernutrition-induced immunodeficiency remain poorly resolved (Bourke et al., 2019, 2016; Hess, 1932; Newsholme, 1908). The primary explanation given for this dysfunction has centered on defects in lymphocyte biology. Patient data and animal studies suggest that T, B, and NK cells are reduced in the periphery of undernourished patients and experimental animals (Campbell et al., 2020; Cason et al., 1986; Contreras et al., 2018; Howard et al., 1999; Nájera et al., 2004; Saha et al., 1977; Schattner et al., 1990; Yang et al., 2009). Moreover, animal models of short-term fasting and protein deficiency have been found to impair T cell expansion, cytokine production, and memory recall responses (Chatraw et al., 2008; Iyer et al., 2012; Mangheri et al., 1992; Procaccini et al., 2012; Saucillo et al, 2014; Taylor et al., 2013). While the contribution of lymphocyte dysfunction to nutritionally acquired immunodeficiency is well established, the impact of prolonged undernutrition on other immune cell populations and the role they play in disease susceptibility is not well understood.

Prior studies have reported disparate impacts of undernutrition on immunity and infection, with some finding restriction enhances inflammation or immunity, while others observing a loss in immune function (Bhattacharjee et al., 2021; Campbell et al., 2020; Chatraw et al., 2008; Collins et al., 2019; Han et al., 2023; Hasegawa et al., 2012; Iyer et al., 2012; Palma et al., 2021; Pena-cruz et al, 1989; Piccio et al., 2008; Starr et al., 2016; Sun et al., 2001; Taylor et al., 2013). These discrepancies likely result from whether animals are subjected to caloric restriction alone or if there is also corresponding restriction of key micro- and macronutrients and whether the restriction is administered as a short-term fast or is sustained (Cerqueira and Kowaltowski, 2010; Contreras et al., 2018; Meydani et al., 2016; Palma et al., 2021; Yan et al., 2021). To this end, caloric and nutrient restriction diets significantly differ in weight loss patterns, physiology, and nutritional state (Cerqueira and Kowaltowski, 2010). While both diets are associated with reduced inflammation, caloric restriction is understood to support both lifespan and health span, in stark contrast to chronic undernutrition (Collins and Belkaid, 2020; Contreras et al., 2018; Goldberg et al., 2015; Green et al., 2021; Hasegawa et al., 2012; Howard et al., 1999; Jordan et al., 2019; Piccio et al., 2008; Spadaro et al., 2022; Sun et al., 2001; Yang et al., 2009).

Beyond our mechanistic understanding of nutritionally acquired immunodeficiency, there is a limited knowledge on whether this dysfunction is reversible (Contreras et al., 2018; Schattner et al., 1990; Vaisman et al., 2004). Because current treatment guidelines for undernourished patients utilize refeeding protocols that focus on weight gain as the indicator of recovery, the effects of refeeding on the immune system are understudied (Ashworth et al., 2003). Moreover, whether prior exposure to malnutrition has durable effects on the ability to control infection following refeeding remains unknown.

Here, we investigate the cellular mechanisms underlying nutritionally acquired immunodeficiency and the ability of refeeding intervention to restore immune function. We employ a chronic murine dietary restriction model and demonstrate that it effectively recapitulates the hallmarks of human undernutrition. We find that chronic undernutrition results in a specific atrophy of immune organs that is not mirrored in essential organs, such as the liver and kidney. This loss of lymphoid tissue is accompanied by a broad reduction in both innate and adaptive immune cell compartments. Undernourished animals fail to control sub-lethal bacterial infections, either succumbing to disease or failing to clear pathogen long-term. We find that while these animals undergo the initial phase of immune cell expansion following infection, they fail to sustain it and the response rapidly undergoes contraction. Accordingly, we observe that T cells in these animals display muted expansion, accelerated contraction, and impaired effector function. We further find that undernutrition selectively impairs steady-state and emergency myelopoiesis, resulting in reduced neutrophil and monocyte abundance prior to and post-infection. Finally, we demonstrate that while refeeding protocols are sufficient to restore body mass, growth, and reverse global lymphoid atrophy, refed animals remain more susceptible to bacterial infection even months after recovering size and peripheral immune cell numbers. In contrast to the ability of refeeding to reverse the effects of undernutrition on lymphoid numbers, we go on to show refed mice display impaired emergency myelopoiesis as well as neutrophil and monocyte abundance. Altogether, our work highlights the cellular mechanisms underlying increased susceptibility to infection during chronic undernutrition, identifying dysregulated myelopoiesis as a major driver. Furthermore, we for the first time demonstrate that refeeding protocols are not sufficient to reverse defects in the ability of animals to control bacterial infection or dysregulated myelopoiesis, despite outwardly displaying a recovery from malnutrition. We believe these findings have important implications for global public health policy and medical standards of care not only for treating people actively experiencing chronic malnutrition, but also for individuals who have experienced and recovered from food scarcity as part of their life history.

## Results

### Sustained dietary restriction recapitulates the hallmarks of nutritionally acquired immunodeficiency

**Figure 1.**
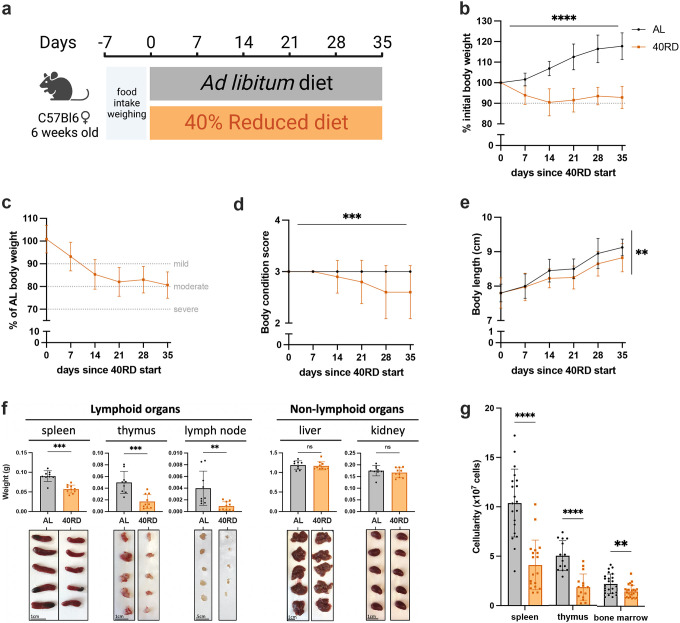
Sustained dietary restriction recapitulates the hallmarks of nutritionally acquired immunodeficiency. (a) Schematic of experimental design for 40% reduced diet (40RD, organge) in comparison to control *ad libitum* (AL, black) diet. (b) Body weight of AL and 40RD mice as a percentage of initial body weight over time (n=50). The dotted line represents 10% of initial body weight lost. (c) Body weight of 40RD mice as a percentage of age matched average AL body weight over time (n=50). Each dotted line represents clinical designations of undernutrition severity. (d) Body condition score of AL and 40RD mice over time (n=10). (e) Body length of AL and 40RD mice over time, measured from the nose tip to the base of the tail (n=10). (f) Comparative weights of AL and 40RD lymphoid and non-lymphoid tissues (n=10) with representative photos of the corresponding organs. Scale bars 1 cm (0.5 cm for lymph nodes). (g) Total live cell counts for whole spleen (n=15), thymus (n=15), and bone marrow (n=10). Statistics: (b-g) Plotted as mean ± SD; (b,d) simple linear regression with slope comparisons; (e) simple linear regression with elevation comparison; and (f,g) two-tailed Mann-Whitney test.

To investigate the relationship of undernutrition and immunodeficiency, we began by adopting a faithful model of chronic malnutrition. In patients, undernutrition is associated with: (1) a consistent weight loss of 10% or more compared to the age-appropriate body weight that occurs in under 6 months and persists for months to years; (2) stunting of growth; and (3) a change in body composition associated with weight loss (Institute of Medicine, 2000; Maleta, 2006; Martins et al., 2011). While short-term fasting and macronutrient or caloric restriction models have been previously used, we elected to employ a 40% reduced diet (40RD) model, which provides both the weight loss as well as micro- and macronutrient deficiency over extended periods of time (Cerqueira and Kowaltowski, 2010). For the 40RD system, the average food intake of C57Bl6 mice was measured at baseline consumption. After a week of baseline measurements, mice were randomly assigned to either the *ad libitum* fed (AL) or the 40RD group. 40% of the average food intake by weight was removed from the diet of 40RD animals (Fig. 1a). Animal weight, body length, and body condition score (BCS) were then regularly surveyed to measure wasting, stunting, and body composition, respectively. Highlighting the ability of the model to reliably generate a state of moderate undernutrition, 40RD animals consistently lost 10% of their initial body weight (IBW) in 4 weeks (Fig. 1b) and achieved an average 20% loss of IBW relative to the AL group (Fig. 1c). At the same time, BCSs decreased significantly in 40RD animals compared to the AL group over the course of 4 weeks (Fig. 1d). This decrease in weight and conditioning was further accompanied by delayed growth and stunting in 40RD animals, as measured by body length (Fig. 1e).

Beyond these gross changes in body condition, undernourished patients also exhibit severe lymphoid organ atrophy (Beisel, 1996; Bourke et al., 2016b). In keeping with this, we observed a reduction in spleen, thymus, and lymph node size and mass in mice given the 40RD diet, while organs such as the liver and kidney were unchanged, suggesting a specific effect of malnutrition on the immune system (Fig. 1f). We observed a corresponding reduction in the cellularity of the spleen and thymus, while the cellularity of the bone marrow was unaffected (Fig. 1g). Collectively, our findings demonstrate that the 40RD model reproduces the lymphoid atrophy found in chronically undernourished patients as well as other models of undernutrition in rodents (Beisel, 1996; Bourke et al., 2016b; Cason et al., 1986; Contreras et al., 2018; Yang et al., 2009).

### Chronic malnutrition results in a failure to control sub-lethal bacterial infection

**Figure 2.**
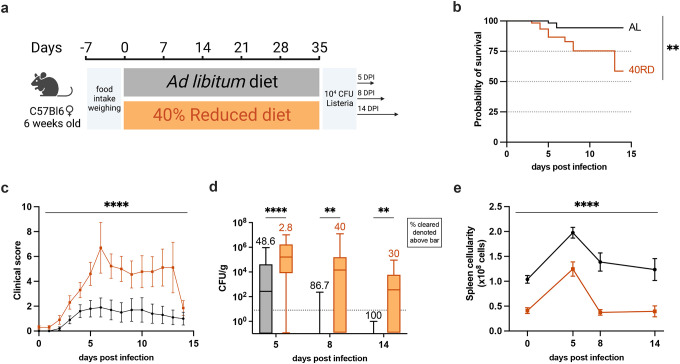
Chronic malnutrition results in a failure to control sub-lethal bacterial infection. (a) Schematic of Lm infection (10^4^ CFUs per mouse) experimental deseign in AL (orange) and 40RD (black) mice. Mice were maintained on the corresponding diet throughout the course of the infection. (b) Probability of survival for infected AL and 40RD mice over time. The curves represent pooled data from 3 experimental groups: 5DPI (n=25), 8DPI (n=15), and 14DPI (n=10). Statistics done via log-rank test. (c) Clinical score for infected AL and 40RD mice over time from 14DPI group. Plotted as mean ± SEM; statistics done via mixed-effect two-way ANOVA analysis. (d) Pathogen burden in liver tissue of AL and 40RD mice. Percentage of mice that cleared the pathogen on a given day is represented as numbers above corresponding bars. The dotted line represents the limit of detection. Plotted as box and min to max whiskers; statistics done via two-tailed Mann-Whitney test for each time point. (e) Total splenocyte counts for infected AL and 40RD mice over time. Uninfected spleen cell counts same as used for Figure 1g. Plotted as mean ± SEM; statistics done via mixed-effect two-way ANOVA analysis.

We next sought to investigate how malnutrition impacted immune responses to infection. While the link between poor infection outcomes and undernutrition has been extensively documented, the immunologic mechanisms resulting in poor infection resolution remain unknown (Bourke et al., 2019, 2016b; Dubos, 1955; Ishikawa et al., 2012; Rice et al., 2000b). To address this, we turned to the Ovalbumin expressing *Listeria monocytogenes* (Lm-Ova) system, a classic model of bacterial infection that permits the tracking of adaptive immunity, innate immunity, and bacterial burden. Both 40RD and AL animals were infected with a sublethal dose of Lm-Ova and then infection progression, pathogen clearance, and immune responses were assessed at 5, 8, and 14 days post-infection (Fig. 2a). Whereas all animals in the AL group survived, nearly half of the 40RD animals became moribund and required euthanasia (Fig. 2b). Even amongst surviving animals, the 40RD group displayed higher clinical scores than the AL group throughout the course of infection (Fig. 2c). Consistent with this, 40RD animals failed to clear bacteria at the rate of the AL group, with some mice exhibiting persistent infection (Fig. 2d). Amongst the AL group, 48.6% and 86.7% of the animals were able to clear bacteria by day 5 and day 8 post-infection, respectively, with all animals resolving infection by day 14. In contrast, 40RD animals exhibited delayed clearance kinetics with only a third of the animals becoming pathogen-free by day 14 (Fig. 2d).

These observations prompted us to ask whether 40RD mice were capable of mounting a normal immune response to infection. To begin addressing this, we examined immune cell expansion in AL and 40RD mice throughout the course of infection. We found that while splenocyte numbers increased at early time points following infection in 40RD mice, the magnitude and duration of this expansion was significantly lower compared to AL controls (Fig. 2e). Thus, chronic malnutrition is sufficient to not only induce immune atrophy, but further abrogates the capacity of animals to mount and sustain an immune response to infection.

### Chronic malnutrition diminishes T cell expansion and function while accelerating contraction during infection

Considering the broad defects observed in infected, chronically undernourished animals, we next aimed to investigate the effects malnutrition had on specific immune cell populations during this response. As malnourished patients are known to exhibit the reduction in lymphocyte numbers, we assessed whether 40RD animals exhibited signs of lymphopenia following infection (Cason et al., 1986; Nájera et al., 2004; Saha et al., 1977). We observed that both prior to and after infection, 40RD animals displayed reduced numbers of B cells, CD4 T cells, and CD8 T cells compared to controls (Supplemental 1). Despite this overall loss in lymphocyte number, the relative frequency of each population was either unchanged or elevated, indicating that while malnutrition leads to a global reduction in immune cell numbers, lymphocytes are less impacted than other immune cell populations (Supplemental 1).

**Figure 3.**
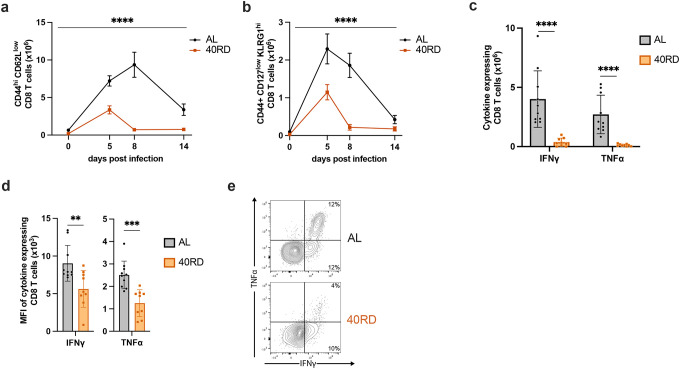
Chronic malnutrition diminishes T cell expansion and function while accelerating contraction during infection. AL and 40RD mice were infected with Lm at 10^4^ CFUs per mouse. Splenocytes were counted and flow cytometry was performed at days 0, 5, 8, and 14 post-infection to evaluate the total cell number of (a) antigen-experienced CD8^+^ T cells gated on live single CD8^+^ CD44^hi^ CD62L^low^ and (b) short lived effector cells further gated on KLRG1^hi^ CD127^low^. Plotted as mean ± SEM; statistics done via mixed-effect two-way ANOVA analysis. Splenocytes from AL (n=10) and 40RD (n=10) mice were harvest at day 8 post-infection and stimulated ex vivo with OVA peptide for 6 hours. Intracellular flow cytometry was performed to quantify (c) total cell number of and (d) mean fluorescence intensity (MFI) of antigen-experienced CD8^+^ T cells expressing IFNγ and TNFα. Plotted as mean ± SD; statistics done via two-tailed Mann-Whitney test for each cytokine. (e) Representative flow cytometry data of (c,d), with average frequencies shown.

In light of the role CD8 T cells play in resolving intracellular bacterial infections and the failure of 40RD animals to clear infection at later time points, we next examined whether the kinetics of T cell expansion and contraction were similarly impacted. To do so, we tracked T cell function throughout the course of infection, on day 5 (early response), day 8 (peak response), and day 14 (contraction phase) post-infection (Qiu et al., 2018; Zenewicz and Shen, 2007). We found that the antigen-experienced (CD44^+^) and short-lived effector (CD127^low^KLRG1^high^) CD8^+^ T cell numbers were significantly reduced throughout the infection (Fig. 3a,b). These effector-like populations expanded minimally during early and peak response and contracted quickly by day 14, prior to pathogen clearance in 40RD animals. In addition to these defects in expansion/contraction dynamics, we further tested whether chronic malnutrition also impaired T cells function. We thus evaluated both the frequency and per-cell cytokine producing capacity of T cells at day 8 post-infection. We observed that T cells from undernourished mice displayed both a decreased frequency of cytokine producing cells and a decreased capacity for producing cytokines on a per-cell basis (Fig. 3c–e). Altogether, these findings suggest that impaired T cell function along with insufficient T cell expansion and premature contraction prior to pathogen clearance contribute to the failure of chronically undernourished animals in controlling bacterial infection.

### Chronically malnourished animals display dysregulated myelopoiesis

**Figure 4.**
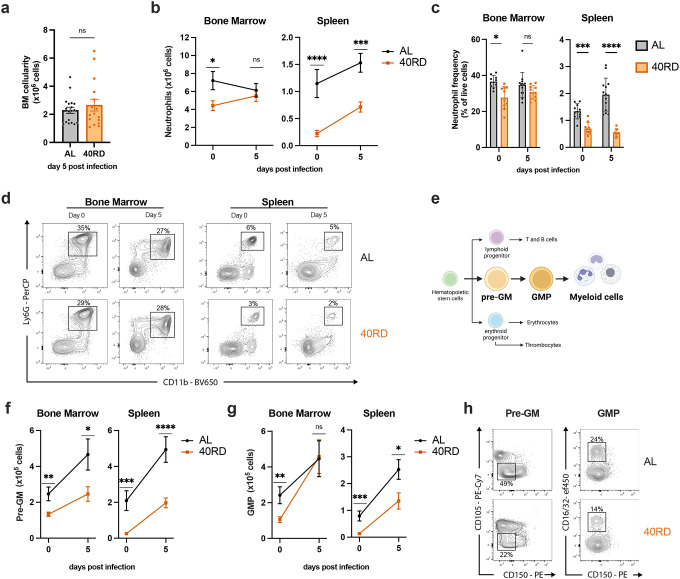
Chronically malnourished animals display dysregulated myelopoiesis. (a) Total bone marrow cell counts from day 5 post-infection AL (n=18) and 40RD (n=16) mice. Bone marrow cells and splenocytes were counted and flow cytometry was performed at days 0 (n=10 for both AL and 40RD) and 5 post Lm infection in AL and 40 RD mice to evaluate (b) the total cell number of neutrophils and (c) the relative frequency of neutrophils among live cells. (d) Representative flow cytometry data for the results in (b,c), with average frequencies shown. (e) A simplified schematic representation of myelopoiesis showing pre-GM and GMP as key progenitors in granulocyte/monocyte lineage. Bone marrow cells and splenocytes were counted and flow cytometry was performed at days 0 and 5 post Lm infection in AL and 40 RD mice to evaluate the total cell number of (f) pre-GM cells (Lineage^−^ Sca1^−^ CD117^+^ CD150^−^ CD16/32^−^ CD105^−^) and (g) GMP cells (Lineage^−^ Sca1^−^ CD117^+^ CD150^−^ CD16/32^+^). (h) Representative flow cytometry results for the myeloid progenitor data in (f,g), with average frequencies shown. Statistics: (a-c,f,g) Plotted as mean ± SEM; statistics done via two-tailed Mann-Whitney test for each time point.

The observation that 40RD animals exhibited elevated pathogen burden at early time points prior to the peak of the adaptive immune response prompted us to investigate whether innate immune responses were similarly impaired. While total bone marrow cellularity was equivalent between 40RD and AL groups, examination of specific innate immune populations in bone marrow revealed that steady-state neutrophil and monocyte levels were significantly lower in undernourished animals, both in absolute numbers and relative abundance (Fig. 1g & Fig. 4a–d; Supplemental 2). In keeping with this, we observed that splenic neutrophil and monocyte abundance were significantly lower in 40RD animals at steady-state and underwent impaired expansion following infection (Fig. 4b–d; Supplemental 2).

Neutrophil and monocyte production is maintained in the bone marrow during steady-state and through emergency myelopoiesis in both bone marrow and spleen upon infection (Janssen et al., 2016; Kim, 2010; Manz and Boettcher, 2014; Yvan-Charvet and Ng, 2019). We thus evaluated how undernutrition affected the key myeloid progenitor populations, pre-granulocyte/monocytes (pre-GM) and granulocyte/monocyte progenitors (GMP) (Fig. 4e). Consistent with a loss of mature neutrophils and monocytes, we found that myelopoiesis was significantly impaired in undernourished mice. Within the bone marrow, pre-GM and GMP cells were present at lower numbers in steady-state 40RD mice (Fig. 4f,g). Upon infection, these bone marrow progenitor populations were capable of undergoing expansion, with pre-GM cells failing to reach AL levels whereas GMP cells reached comparable numbers (Fig. 4f–h). These defects were more exaggerated in the spleen, where 40RD mice displayed diminished numbers and frequency of pre-GM and GMP cells both pre- and post-infection (Fig. 4f–h). Altogether, we find that undernutrition impairs steady-state myelopoiesis and extramedullary emergency myelopoiesis, blunting the innate immune response against a bacterial pathogen.

### Refeeding intervention reverses wasting, stunting, and global immune atrophy

**Figure 5.**
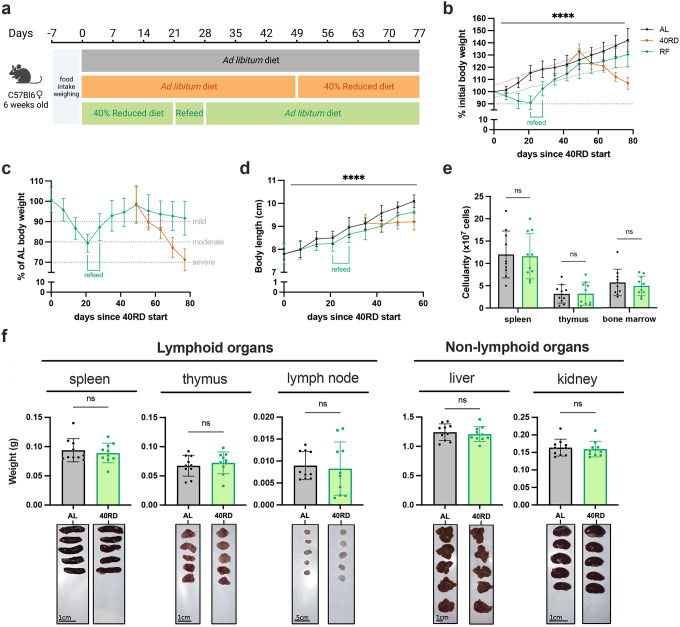
Refeeding intervention reverses wasting, stunting, and global immune atrophy. (a) Schematic of the experimental design for refeeding intervention (RF) in comparison to age-matched 40RD and control AL diet. (b) Body weight of AL (n=25), 40RD (n=10), and RF (n=25) mice as a percentage of their initial body weight over time. The dotted line represents 10% of initial body weight lost, and the shaded area represents the normal weight range for age-matched female C57Bl6 mice(C). (c) Body weight of 40RD (n=10) and RF (n=25) mice as a percentage of age matched average AL body weight over time. Each dotted line represents clinical designations of undernutrition severity. (d) Body length of AL and 40RD mice over time, measured from the nose tip to the base of the tail (n=10). (e) Total cell counts for whole spleen, thymus, and bone marrow from AL and RF mice (n=10). (f) Comparative weights of AL and RF lymphoid and non-lymphoid tissues (n=10) with representative photos of the corresponding organs. Scale bars 1 cm (0.5 cm for lymph nodes). Statistics: (b-f) Plotted as mean ± SD; (b,d) simple linear regression with slope comparisons; and (e,f) two-tailed Mann-Whitney test.

One of the primary strategies taken to support undernourished patients with health complications, including infections, is to refeed them by slowly increasing caloric intake to levels expected for their age group (Ashworth et al., 2003). Despite the widespread employment, the effects of this intervention on immune dysfunction are not known. Following medically recommended standards of care for undernourished patients, we developed a refeeding protocol that safely reintroduces *ad libitum* feeding to 40RD animals to test whether defects in immune responses can be rescued through weight gain and nutrient availability (Ashworth et al., 2003; Mehanna et al., 2008). Undernourished mice undergoing a refeeding protocol (RF) were maintained on a standard 40RD diet for 4 weeks or until 10% BWL. Then, the RF animals were given 10% extra food by weight every two days for a week. On the last day of the refeeding protocol, the RF animals were given *ad libitum* access to feed. Animals were maintained for an additional 6–8 weeks on the *ad libitum* feed until they reached a normal weight range for their age (Fig. 5a). The RF group was able to regain IBW during the refeed period and continued to gain weight at an accelerated pace for the first month (Fig. 5b). After the initial increase in weight, the weight gain pace of RF animals slowed down to the same rate as the AL group (Fig. 5c). We further observed that while RF mice were able to recover growth, the animals maintained a modest but significantly shorter body length than AL controls for their age (Fig. 5d), in keeping with persistent stunting common in undernourished patients (Ashworth et al., 2003).

With the refeeding protocol established, we set out to test whether restoring body mass was sufficient to reverse the lymphoid atrophy found in 40RD animals. Paralleling recovery in weight, we found that cellularity in the spleen, thymus, and bone marrow were comparable between AL and RF groups (Fig. 5e). Moreover, we observed that the weight of lymphoid organs has returned to the AL levels in the RF group (Fig. 5f). Together, these findings indicate that refeeding intervention is sufficient to restore global lymphoid atrophy.

### Refeeding intervention fails to restore immunocompetency and normal myelopoiesis

**Figure 6.**
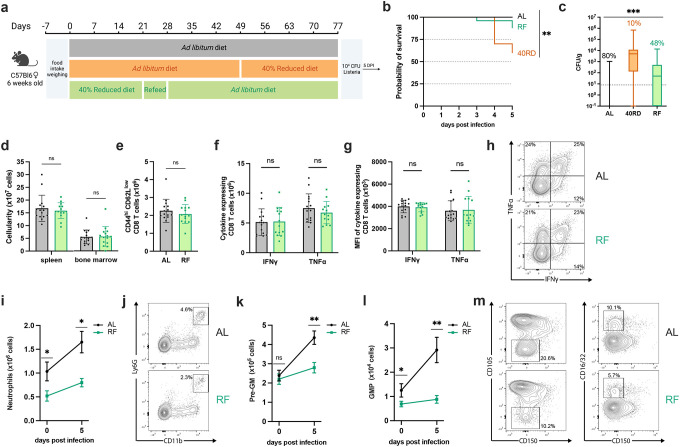
Refeeding intervention fails to restore immunocompetency and normal myelopoiesis. (a) Illustration of Lm infection (10^4^ CFUs per mouse) experimental outline in AL, 40RD, and RF mice. Mice were maintained on the corresponding diet throughout the course of the infection. (b) Probability of survival for infected AL (n=25), 40RD (n=10), and RF (n=25) mice over time. Statistics done via log-rank test. (c) Pathogen burden in liver tissue of 5 DPI AL (n=25), 40RD (n=10), and RF (n=25) mice. Percentage of mice that cleared the pathogen on a given day is represented as numbers above corresponding bars. The dotted line represents the limit of detection. Plotted as box and min to max whiskers; statistics done via Kruskal-Wallis test. (d) Total splenocyte and bone marrow cell counts for AL (n=15) and RF (n=15) mice at day 5 post-infection. (e) Total cell number of antigen-experienced CD8^+^ T cells in AL and RF mice at days 0 and 5 post-infection. Splenocytes from AL and RF mice were harvest at day 5 post-infection and stimulated ex vivo with OVA peptide for 6 hours. Intracellular flow cytometry was performed to quantify (f) total cell number of and (g) mean fluorescence intensity (MFI) of antigen-experienced CD8^+^ T cells expressing IFNγ and TNFα. (h) Representative flow cytometry results for data in (f,g), with average frequencies shown. (i) Total splenic neutrophils abundance in AL and RF animals at days 0 and 5 post-infection. (j) Representative flow cytometry results for day 5 post-infection neutrophil data in (i), with average frequencies shown. At days 0 and 5 post-infection, spleens from AL and RF were evaluate for the total number of (k) pre-GM cells and (l) GMP cells. (m) Representative flow cytometry results plots for the myeloid progenitor data in (k,l), with average the average frequencies shown. Statistics: (d-g) Plotted as mean ± SD; statistics done via two-tailed Mann-Whitney test for each category. (i,k,l) Plotted as mean ± SEM; statistics done via two-tailed Mann-Whitney test for each time point.

After observing successful reversal of the lymphoid atrophy in RF mice, we tested whether refeeding would be sufficient to restore their ability to control bacterial infection. To test this, we infected age-matched AL, 40RD, and RF animals with a sub-lethal dose of Lm-OVA (Fig. 6a). While refeeding was able to limit morbidity compared to 40RD animals, a portion of RF animals succumbed to infection whereas all AL mice remained viable (Fig. 5b). Consistent with this, we observed that animals with ongoing malnutrition maintained the highest pathogen burdens and that RF animals were unable to resolve infection with the same kinetics as AL mice, with less than half RF animals clearing bacteria (Fig. 5c). These data indicate that despite refeeding being sufficient to reverse malnutrition-induced lymphoid atrophy, prior exposure to chronic undernutrition leads to persistent susceptibility to bacterial infection.

Having observed this durable impairment in immune protection, we next aimed to identify the specific facets of malnutrition-induced immune dysfunction that failed to recover in RF mice. In keeping with the steady-state recovery observed, post-infection splenic and bone marrow cellularity were equivalent between RF and AL animals (Fig. 6d). Moreover, we found that T and B cell numbers were restored in RF mice and all lymphocyte populations underwent comparable expansion to AL controls after infection (Supplemental 3). Similarly, we observed no differences in the number of antigen-experienced T cells in the spleen nor T cell functional capacity (Fig. 6e–g). Thus, refeeding permits recovery of lymphocyte number, lymphocyte expansion capacity, and T cell function in response to infection, suggesting lymphocyte dysfunction is unlikely to be responsible for the persistent susceptibility observed.

In contrast to the restoration of the lymphocyte response, we observed sustained defects in the myeloid compartment. RF animals displayed impaired peripheral expansion of neutrophil and monocyte populations after infection, both by frequency and number, with neutrophil number diminished in steady-state RF animals as well (Fig 6i,j; Supplemental 4a,b). Within the bone marrow, we observed a reduced frequency in steady-state monocyte and neutrophil abundance as well as reduced total neutrophil numbers, however both populations expanded upon infection, reaching control numbers (Supplemental 4c–f). Having seen this, we next asked whether RF animals exhibited a persistent impairment in either central or emergency myelopoiesis. Whereas myeloid progenitor populations were not perturbed in the bone marrow, we found that RF mice exhibited significantly reduced frequency and numbers of splenic pre-GM and GMP cells following bacterial infection, with the GMP population also diminished in steady-state animals (Fig. 6j–l); Supplemental 5). Altogether, our findings demonstrate that refeeding intervention uncouples a global recovery in lymphoid atrophy from an enduring impairment in emergency myelopoiesis, resulting in lasting susceptibility to bacterial infection.

## Discussion

Here we employ a murine model of chronic undernutrition that phenocopies the hallmarks of human undernutrition (stunting, wasting, and changes in body composition) to elucidate the cellular mechanisms underlying nutritionally acquired immunodeficiency. We demonstrate that sustained malnutrition results in global lymphoid atrophy, with loss of both innate and adaptive immune cell populations. Additionally, we identified a unique and severe impairment in neutrophil and monocyte abundance as well as myelopoiesis. Our data suggests that the combination of diminished homeostatic levels of these critical myeloid populations along with impaired emergency myelopoiesis leads to poor control of early infection, while a blunted T cell response to infection and their premature contraction results in lack of effective infection resolution. We then tested whether traditional refeeding interventions are effective in restoring protective immunity to infection. We found that restoring food access permitted animals to regain weight and size as well as reverse many signs of immunodeficiency. Refeeding was sufficient to recover lymphoid organ atrophy, the abundance of most circulating lymphocytes, and T cell cytokine production. Nonetheless, refed animals continued to display increased susceptibility to infection along with sustained defects in neutrophil and monocyte abundance and emergency myelopoiesis. These data demonstrate that exposure to chronic undernutrition can result in lasting changes to specific compartments of the immune system, with long-term implications of the health of the individual even after recovering growth and size.

Previous studies have suggested that impaired pathogen responses during undernutrition are driven by lymphocyte loss and functional impairment (Beisel, 1996, 1982; Institute of Medicine, 2000). Low levels of circulating lymphocytes have been well documented in undernourished patients and various restrictive diet models (Campbell et al., 2020; Cason et al., 1986; Contreras et al., 2018; Howard et al., 1999; Saha et al., 1977; Schattner et al., 1990; Yang et al., 2009). Our data from the refeeding model suggest that while these defects likely contribute to poor resolution of infection, they are not sufficient to explain why previously malnourished animals remain vulnerable to infection, as these animals recover from lymphoid atrophy and display normal T cell responses. Nonetheless, we find consistent with other groups that undernourished animals exhibit a steady-state reduction in circulating T cells. Upon infection, the T cell compartment is capable of undergoing expansion in malnourished conditions, but the magnitude of this response is blunted and quickly contracts prior to pathogen clearance. We also found that there was reduced abundance of antigen experienced T cells throughout the infection. Additionally, we found that the intrinsic ability of T cells to produce inflammatory cytokines was reduced. In keeping with work demonstrating that T cells from undernourished patients display reduced function, we find that T cells from malnourished animals have impaired cytokine producing capacity post-infection. Altogether, our work supports clinical observations and earlier studies delineating the relationship of undernutrition on lymphoid atrophy and T cell dysfunction and has revealed the reversible nature of these defects after refeeding intervention.

In contrast to the recovery found in the adaptive immune cells after refeeding, our work demonstrates that exposure to chronic periods of malnutrition results in durable defects in the myeloid compartment. We find that undernourished animals display impaired myelopoiesis and a loss in abundance in critical peripheral myeloid populations that does not properly recover upon refeeding. Indeed, some small cohort studies have shown that circulating neutrophil numbers are reduced in undernourished children and that hematopoiesis is impacted in anorexic patients (Kohama et al., 2021; Vaisman et al., 2004). Thus, while hematopoiesis and peripheral immune cell numbers are broadly sensitive to an animal’s ongoing nutritional status, specific subsets are uniquely sensitive to periods of undernutrition and exhibit nutritional scarring that uncouples their activity from future improvements in dietary intake. These findings raise the possibility that lasting epigenetic changes within the myeloid compartment occur during exposure to undernutrition, akin to what has been observed for trained immunity (Netea et al., 2020). Indeed, a recent study in a small cohort of undernourished children reported changes in H3 acetylation in peripheral blood mononuclear cells (Kupkova et al., 2021).

Altogether our work offers new insight into cellular mechanisms underlying nutritionally acquired immunodeficiency and the ability of refeeding intervention to treat these defects. The burden of morbidity and mortality of infections on undernourished individuals is one of the leading global health crises and evidence of impaired vaccine responses in undernourished people is putting in question the world’s ability to protect the most vulnerable populations with its most reliable strategies for prevention (Bhattacharjee et al., 2021; Bhargava, 2016; Bourke et al., 2019, 2016; Dubos, 1955; Ishikawa et al., 2012; Prendergast, 2015; Rice et al., 2000; Saha et al., 1977). We provide evidence that even prior exposure to food scarcity may be sufficient to permanently alter future immune responses. This suggests that the current dietary status of an individual may be insufficient information to understand the impact nutrition has on their immune system. Future work delineating the specific components of diet required to sustain immune health in the face of food scarcity will be critical for developing nutritional interventions or broader food supplementation programs for at risk populations. We believe that this work highlights the need to account for periods of poverty and food insecurity as a part of patient medical history as well as for formulating global health policy in these areas.

## Materials and Methods

### Mice and diets

All mice were female on a C57Bl6 background maintained at Children’s Hospital of Philadelphia (CHOP) animal facility. Mice were either purchased from Jackson laboratories at 6 weeks of age and acclimated in the CHOP facility for 1 week or bred in house from breeders also obtained from Jackson laboratories. All animal experiments were approved by IACUC, and mice were cared for in accordance with IAC 21-001325 protocol. All 40RD and RF experiments were performed with mice between 6 and 8 weeks of age. Animals were housed in groups of 4–5 animals for all experiments. All experiments were performed on age-matched littermate controls. All animals were maintained on LabDiet 5001 from weaning/arrival to facility.

For the 40RD diet, mice were maintained on *ad libitum* diet until the start of the experiment. One week before the start of the experiment average food intake was measured per 5 mice per day. Average food consumption for all experimental animals between 6 and 8 weeks of age was 4 g/day/mouse. At the start of the experiment, mice were weighed to make sure that all the animals were in the range of ±1.5 g and randomly assigned to an AL or a 40RD group. AL mice were maintained as before with unlimited access to chow. 40RD mice were placed on the 40% reduced diet by weight, resulting in 2.4 g/day/mouse of feed. Consistent weight loss and no competition for food was observed in 40RD mice. 40RD animals lost 10% of their IBW every 4 weeks. If animals lost over 20% of their IBW they were euthanized. During the 40RD diet, experimental animals did not exhibit behavioral or clinical changes and only moderate changed body composition and growth stunting. When all 40RD mice reached 10%−15% IBW loss, they were considered undernourished and utilized for further experiments.

For the RF diet, mice were set up in the identical way to the 40RD diet until they reached 10%−15% IBW loss. At which point they underwent a refeeding intervention developed using Guidelines for the inpatient treatment of severely malnourished children. The refeeding intervention lasted a week and increased the weight of available food to the restricted mice by 10% every 2 days. On the 40RD diet, mice had access to 2.4 g/day/mouse (60% of the initial average food consumption). On day 1 of refeeding intervention, mice had access to 2.8 g/day/mouse (70%). On day 3, they had access to 3.2 g/day/mouse (80%). On day 5, it was 3.6 g/day/mouse (90%). On day 7, mice were given unrestricted food access. RF mice were then maintained on *ad libitum* feed for a month after they entered normal weight range for their age^88^ and their food intake normalized to the AL age-matched controls. At that point mice were considered refed and were utilized for further experiments.

40RD mice and mice on the 1 week-long refeeding intervention were fed daily between 4pm and 6pm to avoid interference with circadian rhythm. Mice on the 40RD and intervention consumed all food provided to them between feeding windows. All mice were observed daily for signs of sickness. All animals were weighed every 2–3 days for 3 times a week total. Food intake for all mice was recorded either daily, if fed daily, or every 2–3 days for 3 times a week total if fed *ad libitum*. Mice were maintained on assigned diets during infection and sepsis experiments. Mice had unlimited access to drinkable water.

### Body length, clinical score, and body condition score (BCS)

For 30 40RD mice and 30 age-matched AL controls body length and BCS were recorded weekly. For 25 RF mice and 25 age-matched AL controls body length were recorded weekly. Body length was recorded using standard centimeter ruler from base of the tail to the tip of the nose. The length was recorded in 0.25cm increments. BCS was recorded using IACUC Pain and Distress Recognition Policy guidelines in a blinded manner by the same observer for all time points. BCS was rated on the scale of 6 ranging from 1 - emaciated to 6 - obese.

Clinical score is a sum of 7 parameters ranked on the scale of 0 - normal to 4 - severely impaired. The scores were measured by the same observer at all time points in a blinded manner. For the measurement, an animal was transferred to a new empty cage and allowed to acclimate for 30 seconds. Then, appearance of the body, appearance of the eyes, level of consciousness, and activity were observed. Then, the cage was tapped twice for auditory stimulus and the animal was touched gently on the back for touch stimulus or gently tipped over if no locomotion was observed. Then, the animal was restrained with standard hold, allowed to acclimate for 30 seconds, and its breathing rate and quality were observed and recorded.

### Listeria preparation and infection

For all the *Listeria monocytogenes* (Lm) infections, the recombinant Lm strain Lm-OVA, kindly provided by the laboratory of Dr. Hao Shen, was used. Lm were grown to early log phase (OD600 = 0.1) in brain heart infusion media (BHI, BD, cat. 237200) at 37°C, washed in PBS (Corning, cat. 21030CV) and diluted to 10^4^ CFUs per 100uL of PBS using our observation that 1 OD unit = 3×10^9^ CFUs. The CFU count was confirmed after infection by plating an aliquot of diluted Lm on BHI plate and incubating overnight at 37°C. Experimental animals were temporarily sedated using Isoflurane (Med Vet International, cat. RXISO-250). 100uL of Lm suspension was injected retro-orbitally per mouse. Mice were observed for full anesthesia recovery. All mice were observed for signs of sickness during infection progression. Any animals passed before the endpoint were tested for CFUs in their liver and recorded as “succumbed to infection” if CFUs were detected or “censored” if CFUs were not detected. For 10 40RD mice and 10 age-matched AL controls, clinical scores were recorded daily at the time of infection and for 14 days following infection.

### Tissue collection and processing

At the end of the experiment, mice were euthanized by carbon dioxide and subsequent cervical dislocation. Either all or a subset of the following tissues were collected, depending on the type of experiment: spleen, bone marrow, thymus, liver, kidney, and inguinal lymph nodes. For steady state assessment, all the tissues were weighed and photographed except for bone marrow before being processed. Liver and kidney were then disposed of and only spleen, thymus, and bone marrow were processed. For the infection model, only spleen and bone marrow were processed, and liver was used for CFU counting described in the next section. Bone marrow was harvested and homogenized by flushing bones with 25G needle and 10mL of cold PBS. Thymus and spleen were harvested in full, homogenized in PBS and strained through a 70-micron cell filter (Celltreat, cat. 229483). All homogenates were resuspended in 1mL of ACK buffer (Quality Biological, cat. 118-156-101) for 2 minutes to perform red blood cell lysis. The reaction was quenched with 10mL of RPMI 1640 supplemented with 10% FBS (Fisher Scientific, cat. BP9703100), 1% Penicillin-streptomycin (Mediatech, cat. MT30-002-CI), 1% L-Glutamate (Mediatech, cat. 1249), 10mM HEPES (Invitrogen, cat. 15630080), 1mM sodium pyruvate (CCS, cat. 1175), and 0.1% 2-Mercaptoethanol (Life technologies, cat. 21985023). Cells then were counted using a hemocytometer and TrypanBlue (Corning, cat. 25-900-CI).

### Liver processing and CFU count

Livers were harvested from Lm infected mice at days 5, 8, and 14-post-infection as described above. Left lateral lobes were collected and weighed. Then, the lobes were homogenized in 1mL of PBS using the Bead Lysis Kit (Next Advance, cat. PINK5E100) and Next Advance Bullet Blender 5E machine for 10 minutes at power 8. The homogenates were serially diluted in PBS between 1:5 and 1:200,000 using serial dilution. The dilutions were plated on BHI plates and incubated at 37oC overnight. A plate with a countable number of CFUs (20 – 100 colonies) was picked per sample. The CFUs were counted by eye and using weight of the lobe and dilution factor, CFUs/g of liver were calculated.

### Flow cytometry and cytokine staining

For surface staining, 4×10^6^ cells from spleen, thymus, and bone marrow homogenates were washed with PBS and stained with a LIVE/DEAD^™^ Fixable Aqua Dead Cell Stain (Fisher Scientific, cat. L34966) for 15 minutes at 4°C. Then, cells were washed and stained for 20 minutes at 4°C in 2% Rat Serum (StemCell, cat. 13551) and PBS with the following antibodies based on the type of the panel (details below).

#### Innate immune cells:

anti-MHC II (eBioscience M5/114.15.2, cat. 48-5321-80, 1:200), anti-CD11b (eBioscience M1/70, cat. 64-0112-82), anti-CD19 (BioLegend 6D5, cat. 115506), anti-Ly6G (Biolegend 1A8, cat. 127616), anti-Ly6C (eBioscience HK1.4, cat. 17-5932-82), anti-Siglec F (eBioscience 1RNM44N, cat. 127616), anti-TCRβ (eBioscience H57-597, cat. 61-5961-82), anti-CD11c (eBioscience N418, cat. 61-5961-82), anti-F4/80 (eBioscience BM8, cat. 17-4801-82, 1:200), anti-NK1.1 (eBioscience PK136, cat. 47-5941-82)

#### Myelopoiesis:

anti-C16/32 (eBioscience 93, cat. 14-0161-81), anti-CD3 (eBioscience 145-2c11, cat. 11-0031-85), anti-CD4 (BioLegend GK1.5, cat. 100406), anti-CD8 (Invitrogen 53-6.7, cat. MA1-10303), anti-CD19 (BioLegend 6D5, cat. 115506), anti-CD11b (BioLegend M1/70, cat. 101206), anti-CD11c (eBioscience N418, cat. 11-0114-85), anti-Ter119 (eBioscience TER-110, cat. 11-5921-85), anti-Ly6G/C (eBioscience RB6-8C5, cat. 11-5931-85), anti-B220 (eBioscience RA3-6B2, cat. 11-0452-85), anti-NK1.1 (eBioscience PK136, cat. 11-5941-85), anti-Sca1 (eBioscience D7, cat. 45-5981-82), CD150 (eBioscience mShed 150, cat. 12-1502-82), CD117 (eBioscience 2B8, cat. 61-1171-82), CD105 (eBioscience MJ7/18, cat. 25-1051-82), CD135 (eBioscience A2F10, cat. 17-1351-82), CD48 (BD HM48.1, cat. 561242, 1:200)

#### Effector T cells:

anti-CD4 (eBioscience GK1.5, cat. 64-0041-82), anti-CD8 (Invitrogen 53-6.7, cat. MA1-10303), anti-CD44 (eBioscience IM7, cat. 45-0441-82), anti-CD127 (eBioscience A7R34, cat. 12-1271-82, 1:200), anti-KLRG1 (eBioscience 2F1, cat. 25-5893-82)

After staining, cells were washed with PBS. If cells were extracted from infected animals, they were also fixed for 30 minutes at 4°C using eBiosciences Intracellular Fixation Buffer (Termo, cat. 88-8824-00).

For intracellular cytokine staining, 4×10^6^ splenocytes from infected mice were plated in 100uL of supplemented RPMI 1640 described above in U-bottom tissue culture treated 96-well plates (Celltreat, cat. 229190). Cells were stimulated with 2 μg/mL of SIINFEKL peptide (Anaspec, cat. AS-60193-5) and 1 mg/mL of Brefeldin A (Invitrogen, cat. inh-bfa) added 1 hour after the peptide for a total of 6 hours at 37°C. After incubation, cells were washed with PBS and stained with L/D aqua dye for 15 minutes at 4°C. Then, cells were washed and stained for 20 minutes at 4°C in 2% Rat Serum and PBS with the following antibodies: anti-CD4 (eBioscience GK1.5, cat. 64-0041-82), anti-CD8 (Invitrogen 53-6.7, cat. MA1-10303), anti-CD44 (eBioscience IM7, cat. 45-0441-82). After staining, cells were washed with PBS and fixed for 30 minutes at 4°C using eBiosciences Intracellular Fixation Buffer. Cells then were washed using eBiosciences Intracellular Permeabilization Buffer (Termo, cat. 88-8824-00) and stained for 30 minutes at room temperature n 2% Rat Serum and Permeabilization Buffer with the following antibodies: anti-TNFα (eBioscience MP6-XT22, cat. 12-7321-82), anti-IL-2 (eBioscience MQ1-17H12, cat. 25-7029-42, 1:200), anti-IFNγ (eBioscience XMG1.2, cat. 17-7311-82).

All antibodies used at 1:300 dilution unless otherwise specified. Regardless of staining, all cells were washed with PBS at the end of the protocol and resuspended in PBS to perform flow cytometry. Stained samples were analyzed on a 4 laser CytoFlex by Beckman Coulter using an automated plate reader. Data were analyzed using FlowJo 10.5.3 software.

### Statistics and reproducibility

All experiments were repeated at least 3 independent times. Data was represented either as a mean ± standard deviation (SD) or standard error of mean (SEM). For measurements over time within the same experimental cohort, statistical analysis was done via simple linear regression comparing slopes or elevation. For survival curves, log-rank test was performed. For all other pairwise comparisons, Mann-Whitney test was performed. For all other measurements, statistical analysis was done via non-parametric one-way (Kruskal-Wallis) or two-way (mixed-effect) ANOVA depending on the number of variables. P-values are recorded on the graphs as follows: **** for p < 0.0001; *** for p < 0.001; ** for p < 0.01; * for p ≤ 0.05; ns for p > 0.05. All statistical analysis was performed using GraphPad Prism 9.2.0 software.

## Supplementary Material

1
