# Supplementary material for: Malnutrition drives infection susceptibility and dysregulated myelopoiesis that persists after refeeding intervention
Source: bioRxiv. 2024 Aug 19:2024.08.19.608575. Preprint. [Version 1] doi: 10.1101/2024.08.19.608575 (PMC11370435; doi:10.1101/2024.08.19.608575)

bioRxiv preprint doi: https://doi.org/10.1101/2024.08.19.608575; this version posted August 19, 2024. The copyright holder for this preprint (which was not certified by peer review) is the author/funder, who has granted bioRxiv a license to display the preprint in perpetuity. It is made available under aCC-BY 4.0 International license.

Supplemental Figure Legends

Supplemental 1.
AL and 40RD animals were infected with 10^4 CFUs of *Listeria monocytogenes* per mouse. At days 0 and 5 post-infection, spleens were harvested, counted, and analyzed by flow cytometry. (a) The total number of splenic B cells. (b) Relative abundance of B cells as a percentage of all live cells. (c) The total number of splenic CD4 T cells. (d) Relative abundance of CD4 T cells as a percentage of all live cells. (e) The total number of splenic CD8 T cells. (e) Relative abundance of CD8 T cells as a percentage of all live cells.

Supplemental 2.
AL and 40RD animals were infected with 10^4 CFUs of *Listeria monocytogenes* per mouse. At days 0 and 5 post-infection, bone marrow and spleens were harvested, counted, and analyzed by flow cytometry. (a) The total number of bone marrow monocytes. (b) Relative abundance of bone marrow as a percentage of all live cells. (c) The total number of splenic monocytes. (d) Relative abundance of splenic monocytes as a percentage of all live cells.

Supplemental 3.
AL and RF animals were infected with 10^4 CFUs of *Listeria monocytogenes* per mouse. At days 0 and 5 post-infection, spleens were harvested, counted, and analyzed by flow cytometry. (a) Relative abundance of B cells as a percentage of all live cells. (b) The total number of splenic B cells. (c) Relative abundance of CD4 T cells as a percentage of all live cells. (d) The total number of splenic CD4 T cells. (e) Relative abundance of CD8 T cells as a percentage of all live cells. (f) The total number of splenic CD8 T cells.

Supplemental 4.
AL and RF animals were infected with 10^4 CFUs of *Listeria monocytogenes* per mouse. At days 0 and 5 post-infection, bone marrow and spleens were harvested, counted, and analyzed by flow cytometry. (a) Relative abundance of splenic monocytes as a percentage of all live cells. (b) The total number of splenic monocytes cells. (c) Relative abundance of bone marrow neutrophils as a percentage of all live cells. (d) The total number of bone marrow neutrophils. (e) Relative abundance of bone marrow monocytes as a percentage of all live cells. (f) The total number of bone marrow monocytes.

Supplemental 5.
AL and RF animals were infected with 10^4 CFUs of *Listeria monocytogenes* per mouse. At days 0 and 5 post-infection, bone marrow was harvested, counted, and analyzed by flow cytometry. (a) Relative abundance of pre-GM cells as a percentage of all live cells. (b) The total number of pre-GM cells. (c) Relative abundance of GMP cells as a percentage of all live cells. (d) The total number of GMP cells.

Supplemental 1

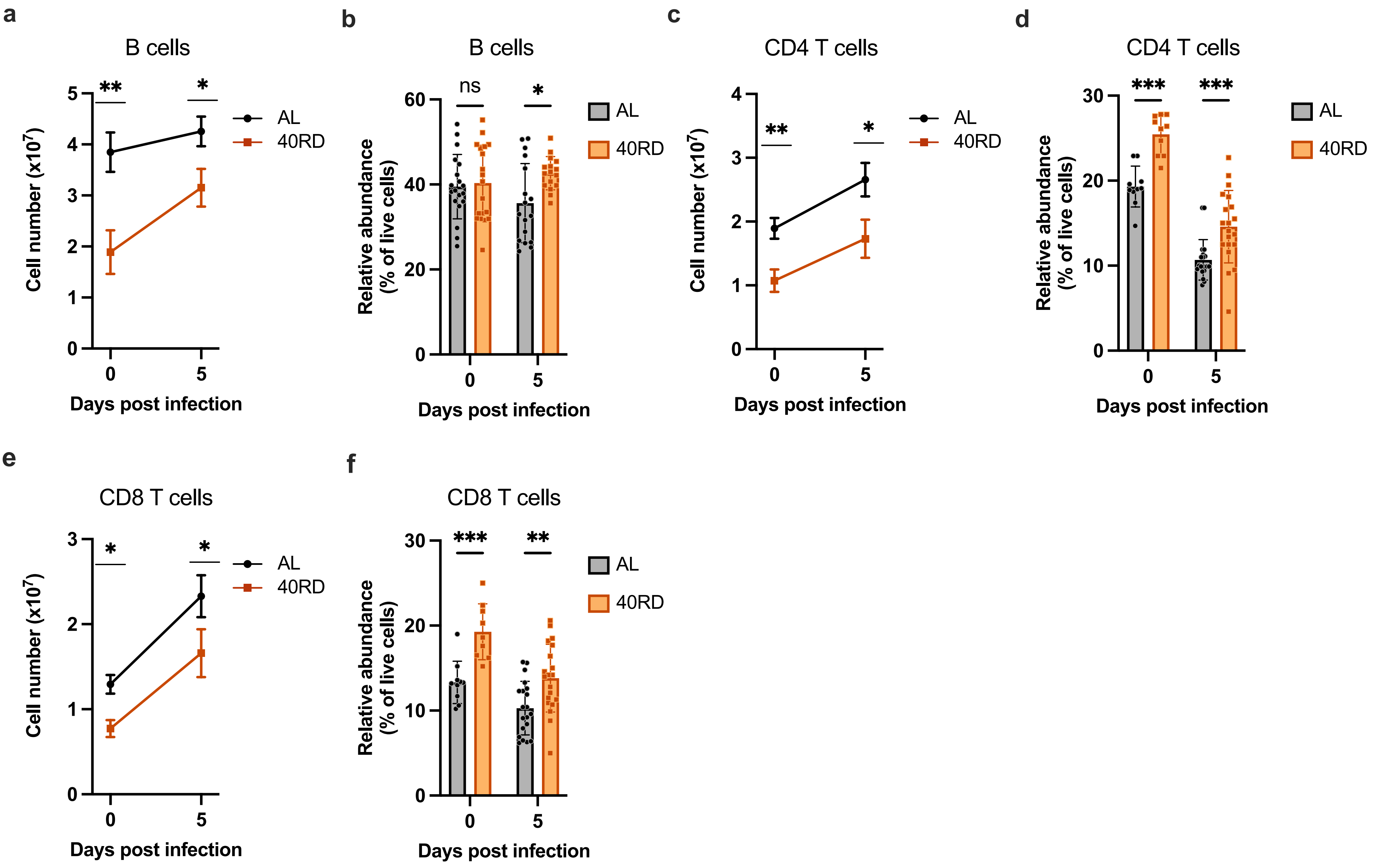

Supplemental 2

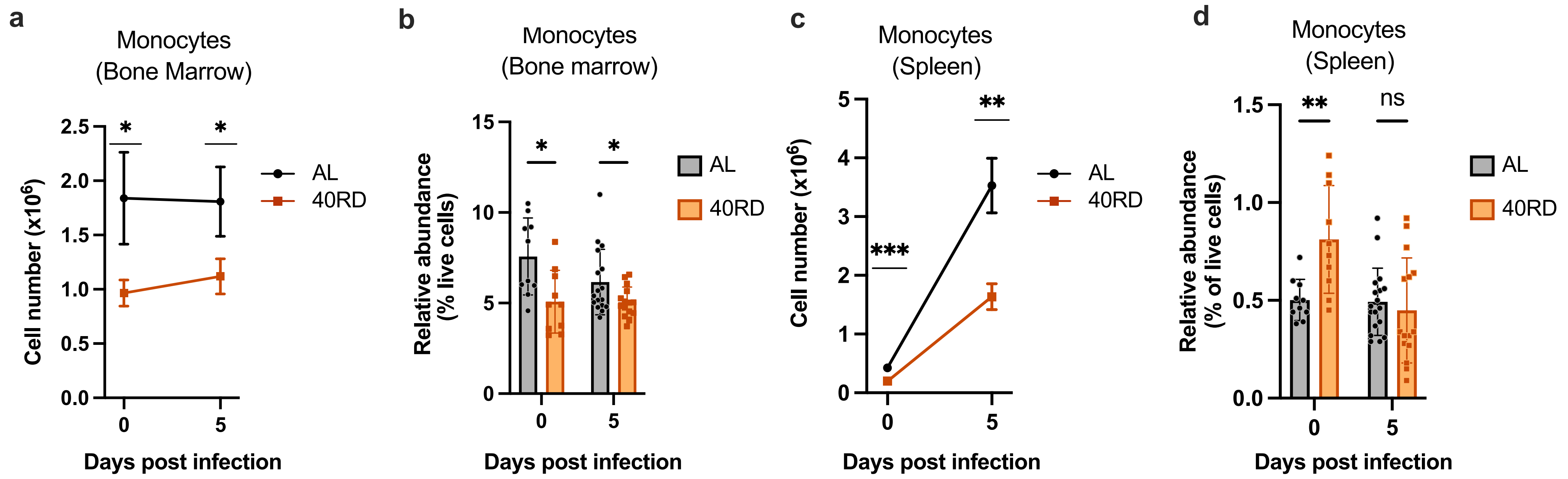

Supplemental 3

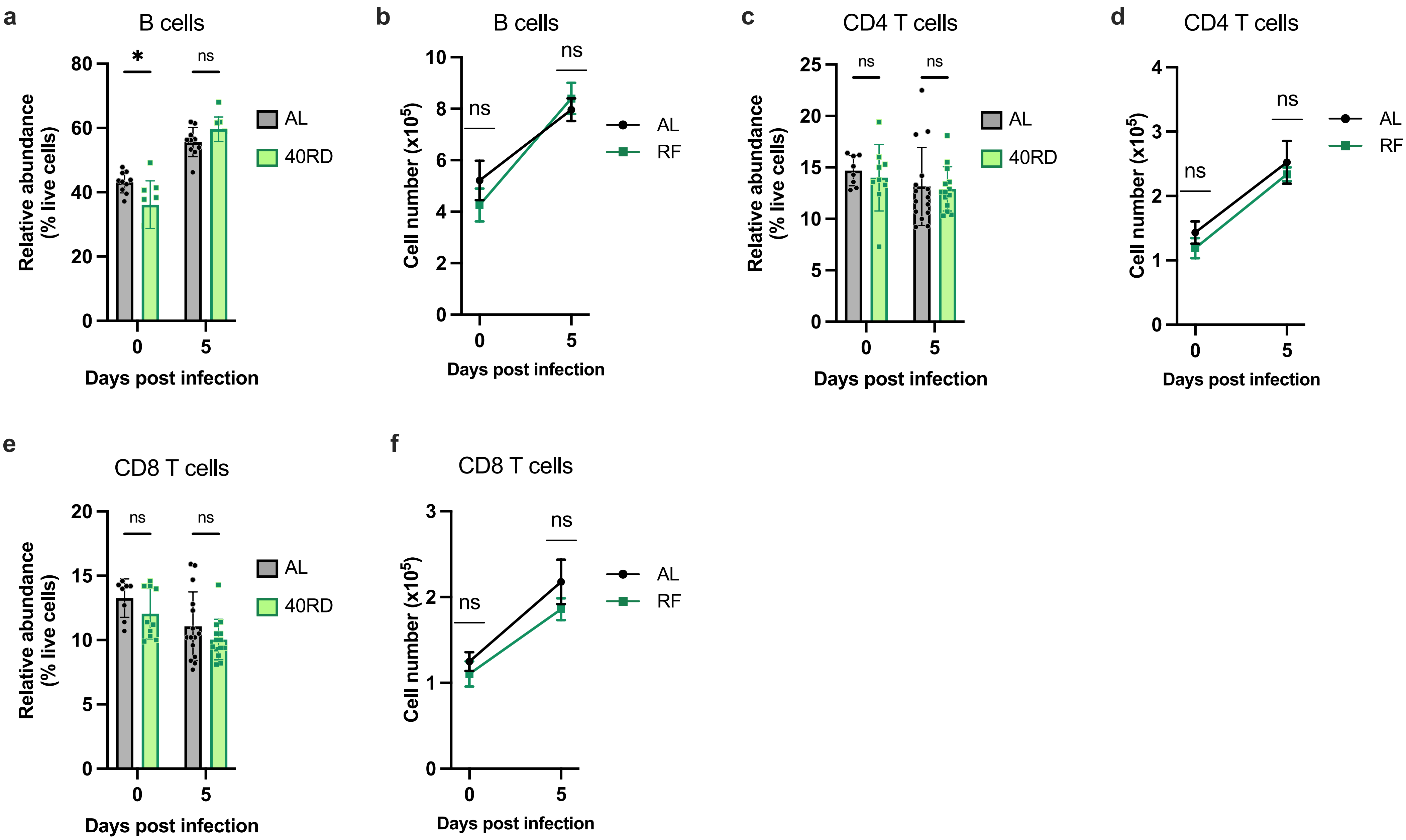

Supplemental 4

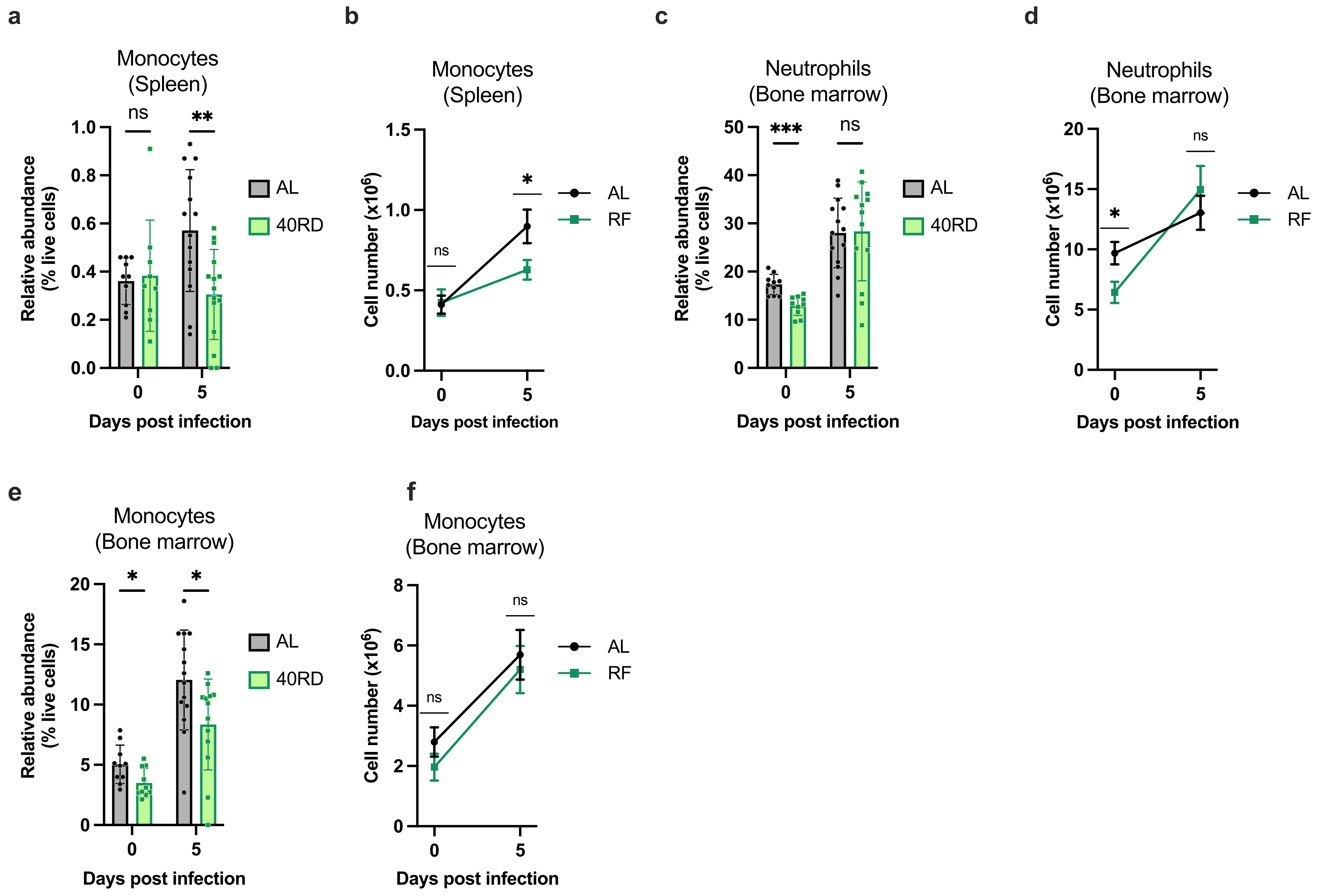

Supplemental 5

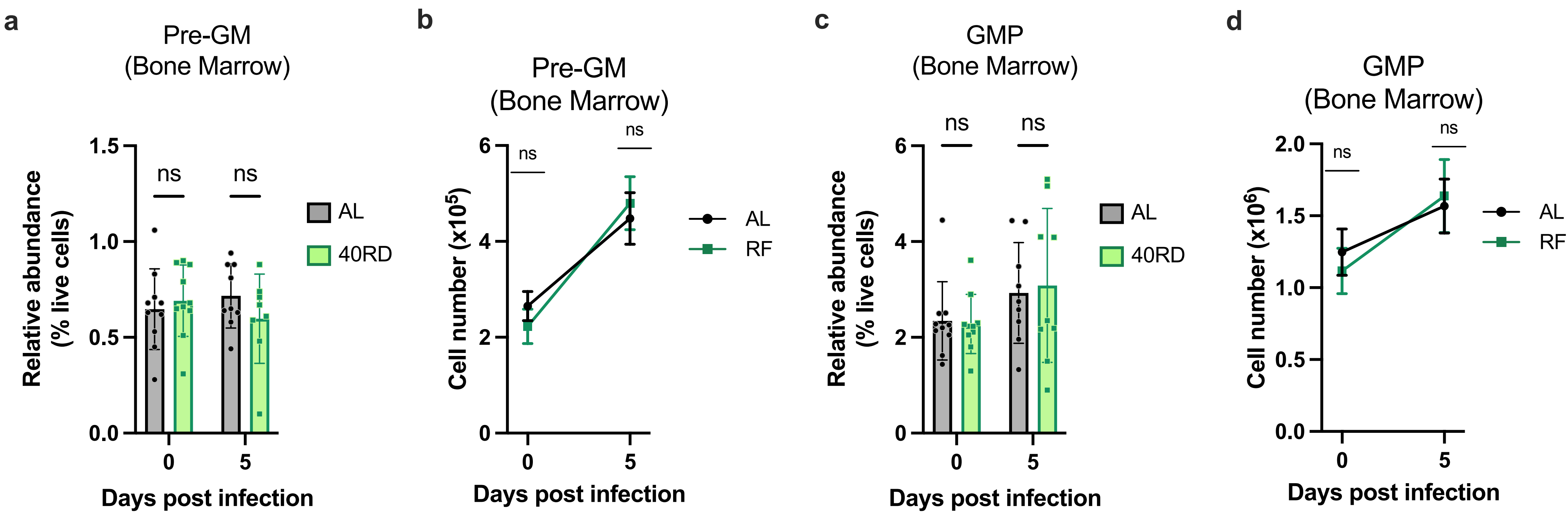

Supplement: Supplementary file 1 [file NIHPP2024.08.19.608575V1-supplement-1.pdf]
